# Regulatory Networks that Direct the Development of Specialized Cell Types in the *Drosophila* Heart

**DOI:** 10.3390/jcdd3020018

**Published:** 2016-05-12

**Authors:** TyAnna L. Lovato, Richard M. Cripps

**Affiliations:** Department of Biology, University of New Mexico, Albuquerque, NM 87131, USA; tyanna@unm.edu

**Keywords:** Drosophila, heart, transcription factor, inflow tract, cardiac valve, alary muscle, outflow tract, Tinman, seven-up

## Abstract

The Drosophila cardiac tube was once thought to be a simple linear structure, however research over the past 15 years has revealed significant cellular and molecular complexity to this organ. Prior reviews have focused upon the gene regulatory networks responsible for the specification of the cardiac field and the activation of cardiac muscle structural genes. Here we focus upon highlighting the existence, function, and development of unique cell types within the dorsal vessel, and discuss their correspondence to analogous structures in the vertebrate heart.

## 1. Introduction

Severe congenital heart defects (CHD) are the most common birth defects that result in death, whereas less severe cardiac defects are often undetected and do not cause problems until much later in life. It is estimated that 1 in 150 adults have some form of congenital heart defect [1]. While the cause of CHD is often unknown, the sequencing of the human genome has led to the discovery of genetic causes in many instances. CHD genetic causes can arise from chromosomal disruptions such as aneuploidy or microdeletions, from single gene mutations associated with a syndrome, or from single gene defects not associated with a syndrome that affect transcription factors or signal transduction pathways functioning in heart development [2].

In addition to genetic factors, heart defects and disease can develop or become exacerbated by external factors such as smoking, or by chronic diseases associated with aging such as diabetes, high blood pressure and hyperlipidemia [3]. Chronic diseases can have genetic as well as lifestyle causes themselves, and interestingly, as they progress they can cause changes in gene expression [4]. When the heart responds to biomechanical stress due to a genetic defect or chronic diseased state, it responds at the molecular level by attempting to remodel itself by re-starting the fetal gene expression pattern [5] which can be measured and is a significant predictor of heart failure. With such a circular combination of genetic and lifestyle contributing factors that affect the function of the heart, an organ that must beat regularly for decades, it is not surprising that heart diseases are the leading cause of death worldwide and that they kill more people than all forms of cancer combined [1]. Clearly, understanding the genetic programs that underpin normal cardiac development will provide critical insight into pathways that are affected in diseased tissues, and potential therapeutic options for their treatment.

At the genetic level, an ever growing collection of conserved regulatory factors contribute to heart specification in all organisms that possess a heart. This suggests that a primitive heart was present in a common ancestor and that it was crafted using a genetic toolbox that has been diversified but not fundamentally changed. As the heart increased in complexity, many of the genes encoding these factors duplicated and their expression patterns became more specialized [6,7]. Nevertheless, the existence of a fundamental cardiac toolbox has meant that model organisms have contributed significantly to our current understanding of heart specification and development.

In particular, *Drosophila* has been a useful model for understanding the components of this toolbox and their genetic interactions, due to a number of factors. First, the embryology of early heart development is conserved between *Drosophila* and vertebrates: during the initial stages of vertebrate heart development, a horseshoe shape of symmetrical cells forms on either side of the embryonic midline from the lateral plate mesoderm. This initial bilateral organization resembles the cardiac precursors in a stage 12 embryo in *Drosophila*. In vertebrates, the cells migrate ventrally to form a muscular linear tube that surrounds an endocardial lining, similar to the dorsal migration of *Drosophila* cardial cells to form a linear beating tube. While Drosophila heart morphogenesis stops at the linear heart tube stage, in humans the linear tube continues to grow rapidly and begins to bend to the right. The left and right ventricles become distinguished from each other at approximately embryonic day 28. The developing heart resembles a fully developed human heart by embryonic day 50 with the left and right atria separated and connected to their corresponding ventricles as well as fully developed inflow and outflow tracts, but it will continue to grow in size until adulthood [8].

Second, *Drosophila* has not undergone genome duplication, and as a consequence null mutations in most of the core factors in *Drosophila* result in distinct cardiac phenotypes. By contrast in vertebrates, a null mutation in a cardiac regulatory factor might not have a severe effect due to compensation from a duplicated gene with overlapping function.

Third, as a result of revolutionary sequencing techniques, it has been determined that of the 1682 human genes identified to have a mutation that causes disease, 74% have an orthologous gene in *Drosophila* [9,10]. Additionally, one third of these genes show functional conservation [11]. More specifically, mutations in many of the genes identified in *Drosophila* heart development have been shown to cause coronary heart disease in humans [12,13,14]. Taken together, these lines of evidence underline the current and future potential for studying cardiac development in Drosophila, as a model for understanding heart development and disease in humans.

The vertebrate heart goes on to become a much more complex pump, however it has a similar function to the *Drosophila* linear pump in that they both serve to distribute nutrients throughout the organism. Moreover, it is now appreciated that the mature Drosophila heart is a complex organ that, like the vertebrate organ, comprises inflow and outflow tracts, valves, and supportive muscle cells. Are the genetic processes that control the formation of specialized cardiac cell types also conserved across large evolutionary distance, in the same manner that cardiac specification factors are conserved? The answer to this question remains open, but emerging data suggest tantalizing similarities in how some of these structures are genetically programmed. Here, we first provide a description of the development and structure of the Drosophila heart, and then present the current understanding of how specialized cell types in the Drosophila heart are formed.

## 2. Development and Organization of the *Drosophila* Dorsal Vessel

**Figure 1:**
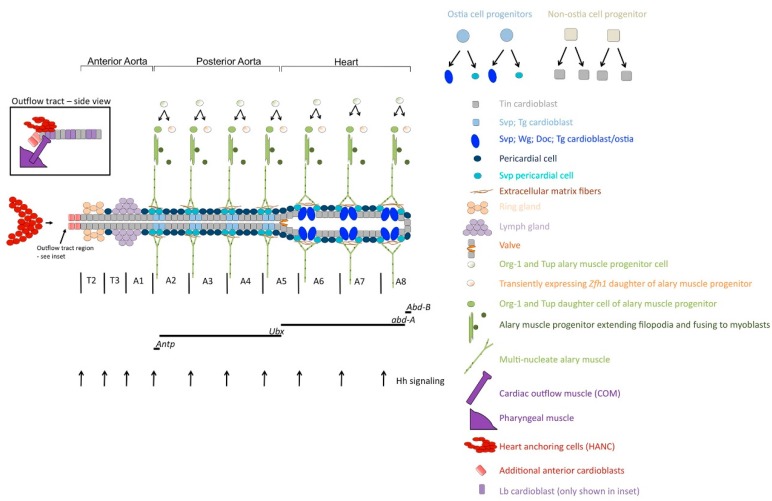
Arrangement and development of the embryonic dorsal vessel.

The *Drosophila* embryonic heart forms from bilateral rows of mesodermal cells that migrate to the dorsal midline to form a linear contractile tube [15] Figure 1. The posterior portion of the tube, called the heart, spans abdominal segments A5 through A8 and has a larger lumen than the more anterior region, or aorta, which spans thoracic segment T2 through A5 [16]. The heart contains six pairs of cells that comprise the ostia, or inflow tracts of the heart. It is through these tracts that hemolymph enters the heart and is pumped anteriorly until it flows out of the aorta, functioning to deliver nutrients throughout the organism. In addition to the myocardial cells of the dorsal vessel, non-contracting pericardial cells surround the muscular cells and act as nephrocytes later in development [17]. Finally, a pair of contractile cells form a cardiovascular valve at the junction of the heart and aorta in the embryonic and larval heart. Live imaging demonstrates that as the heart cells contract, the valve opens to allow hemolymph to enter the aorta; and as the heart cells relax, the cardiovascular valve closes to prevent back flow [18]. Specialized skeletal muscles termed alary muscles also attach near the dorsal side of the heart as well as laterally to the body wall [19,20,21].

During larval development, the cellular organization of the dorsal vessel remains largely unchanged except for an increase in size, but it is significantly remodeled during metamorphosis. During pupariation, the posterior portion of the dorsal vessel is histolyzed, and specialized cells in the aorta develop into ostia. Also during pupariation, multiple myocardial cells appear 48 h after puparium formation (APF) along the ventral surface of the heart and form longitudinal fibers [22,23,24,25,26]. In addition, there are pairs of valves that form in abdominal segments A2, A3 and A4, which separates the heart tube into four separate segments or chambers [20,27]. Clearly, the Drosophila dorsal vessel is a complex and dynamic structure that comprises multiple cell types.

## 3. Cell-Type Diversification in the Dorsal Vessel

At the molecular level, the cardiac tube consists of two distinct cell types, that are characterized by their expression of either the homeobox gene *tinman* (*tin*) or the orphan nuclear receptor gene *seven-up* (*svp*) [15,28,29] Figure 1, and these cells persist throughout development into the adult stage [22]. There are seven sets of Svp cells in the embryonic cardiac tube, corresponding to the seven most posterior cardiac segments, while the three most anterior cardiac segments, termed the anterior aorta, do not contain Svp cells [15]. In the embryonic heart, it is the *svp* expressing cells that comprise the ostia. The two most posterior pairs of larval ostia are histolyzed during pupariation, whereas the anterior four sets of Svp cells define the posterior aorta, and develop into ostia during pupal development [22,24].

**Figure 2:**
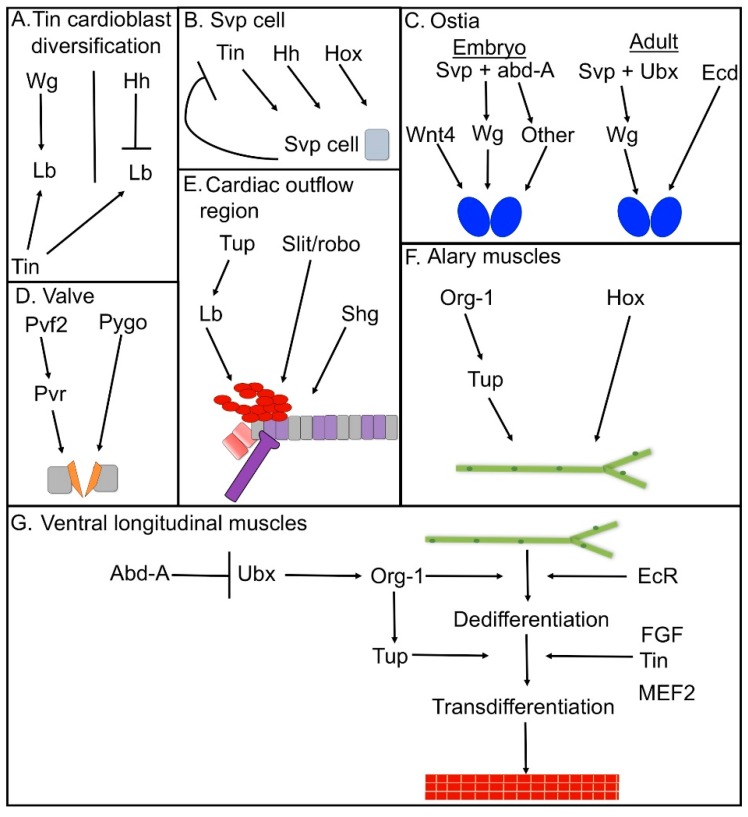
Genes and pathways required for the formation of cardiac structures discussed in this review. (**A**): Diversification of Tin cardioblasts; (**B**): Specification of Svp cells; (**C**): Specification of ostia; (**D**): Specification of the cardiovascular valve; (**E**): Specification of the cardiac outflow region; (**F**): Specification of the alary muscles; (**G**): Formation of the ventral longitudinal muscles of the adult heart.

There is also molecular diversity in the Tin myocardial cells of the dorsal vessel. Jagla *et al*. [30], analyzed the homeobox genes *ladybird-early* (*lb-e*) and *ladybird-late* (*lb-l*), that are expressed in a subset of cardiac precursors directly below the Wg ectodermal stripe, and that have overlapping functions in the specification of a subset of cardial cells. Later in development, Lb protein accumulates in two out of the four Tin cells per hemisegment, providing additional molecular diversity to cells of the dorsal vessel. Lb cell fate is specified by Wingless (Wg) signaling and repressed by Hedgehog (Hh) signaling (Figure 2A), but the functional significance of late Lb accumulation along the cardiac tube is yet to be fully appreciated. However, studies described below demonstrate a requirement for *lb* genes in assembly of the cardiac outflow tract. Interestingly the murine *Lbx1* gene is expressed is a subset of cardiac neural crest cells, and knockout of the gene results in a number of cardiac abnormalities in developing embryos [31].

Progenitors of the Svp cells divide asymmetrically into two *svp* expressing cardioblasts and two *svp* expressing pericardial cells per hemisegment, while the four *tin*-expressing cardioblasts in a given hemisegment arise from the symmetrical division of two non-ostia cell progenitors [32]. The asymmetric division of the ostia cell progenitors is dependent upon *numb* and *sanpodo,* which are part of the *Notch* pathway and have previously been shown to direct asymmetric cell division in other progenitors [33,34]. Specifically, in *sanpodo* mutants duplicate *svp* expressing cardiac cells form, while in *numb* mutants *svp* expressing cardial cells are lost [32,35].

The Hox gene family plays an essential role in directing the differential patterning of the dorsal vessel along the antero-posterior axis of *Drosophila*, and in particular controls the specification of Svp cells. This patterning also ensures that there are functional ostia only in the posterior heart during embryonic and larval stages. The predominant Hox genes include *abdominal-A (abd-A),* which is expressed only in the heart, *Ultrabithorax (Ubx)*, which is expressed in the posterior aorta, and *Antennapedia (Antp)*, which is expressed very narrowly at the junction of the anterior and posterior aorta. When *abd-A is* ectopically expressed, it is sufficient to transform the aorta into a heart, to induce the *svp-*expressing cells of the aorta to form embryonic ostia [36,37,38], and to generate *svp*-expressing cells in the anterior aorta [39]. Ectopic expression of *Ubx* has similar effects, however usually only partial formation of ectopic ostia occurs [36,37]. It is interesting that over-expression of *Ubx* in the aorta, where it is already expressed, results in a similar phenotype as over-expression of *abd-A.* This would suggest that the levels of Hox gene expression play a role in heart cell fate. Finally, over-expression of *Antp* also results in ectopic *svp-*expressing cells [39].

The Hox gain-of-function experiments are supported by loss-of-function studies, demonstrating the requirements of Hox genes for Svp cell specification. Specifically, in loss of function experiments, *abd-A* mutants are the most dramatic in that the heart now resembles the posterior aorta, no ostia form, heart specific markers such as *Tina-1* and *ndae1* are lost, and *Ubx* expression extends into the segments where the heart would have been formed [36,37,38,39]. In *Ubx abd-A* double mutants, most *svp* expression is lost, and for *Antp*, which is only expressed in the most anterior *svp* expressing cells of the aorta, mutants show a loss of *svp* expression only in those two cells [40]. Clearly, antero-posterior identity is a critical determinant of heart cell fate in Drosophila.

Interestingly, *svp* and *tin* are co-expressed at stage 11 of embryogenesis [41] and *tin* is a direct activator of *svp* at this early stage via a conserved cardiac enhancer [40]. However, shortly after the activation of *svp* by Tin, the Tin and Svp cells take on the characteristic, mutually exclusive patterns of expression [35], that presumably arises in large part through mutual cross-repression [41].

Hh signaling has also been shown to play a role in Svp cell specification. Ponzielli *et al.* [37] carried out several experiments to demonstrate the requirement of *hh* for the expression of *svp.* First, they showed a loss of *svp* in an *hh* mutant line, which was rescued by over-expression of *hh* in the ectoderm, but not rescued by expression of a membrane-bound form of Hh. Second, they directed expression in the mesoderm of a repressive *Cubitus interuptus (Ci)* allele, a downstream target of Hh signaling, which partially repressed *svp* expression in the cardiac mesoderm. They then rescued Svp expression in a *wg* mutant background with Hh activity only maintained in the Engrailed-expressing ectodermal cells. Overall, it would appear that while *wg* is required for initial specification of cardioblasts [42], *Hh* is required later for the specification of Svp cells.

Overall, there are three regulatory components that contribute to *svp* expression in the cardiac tube, summarized in Figure 2B): an AP component contributed by the Hox genes; a cardiac context; and a segment polarity component contributed by Hh signaling. Identification of the *svp* cardiac enhancer demonstrated a direct role for Tinman in contributing the cardiac context [43], however how the remaining two sets of signals are integrated at the genomic level has yet to be determined.

A further level of gene function in diversifying the dorsal vessel cells arises from the activities of the T-box genes *Dorsocross 1-3 (Doc1-3), H15,* and *midline (mid)*. The duplicate *Doc* genes have overlapping functions, and they are expressed throughout the cardiac mesoderm in young embryos. Loss of *Doc* results in a complete loss of all cardioblasts [44], underlying the conserved role of T-box genes in cardiac specification [45]. However, at stage 12 *Doc* expression becomes restricted to the Svp cells. Over-expression of *svp* results in expansion of *Doc* in all of the heart cells alongside repression of *tin* and the *Sulfonylurea receptor* (*Sur)* (Lo and Frasch, 2001). Conversely, loss of *svp* results in the loss of *Doc* from the Svp cells and expansion of *tin* and one of its target genes *β3 tubulin* into the Svpcells [35,41].

Loss of the Tbx20-related genes *midline (mid)* and *H15* also affect the pattern of mutually exclusive gene expression in the ostia cells *versus* the contractile cells of the heart, since in *mid* or *mid/H15* deficient mutants, *tin* expression is lost and *Doc* expression expands [46]. A working model for the cross-regulatory interactions of these critical cardiogenic factors was developed by Zaffran *et al.* [47]: *tin* expression in the myocardial cells activated by H15 suppresses *Doc* expression, whereas both Svp and Doc can suppress *tin* expression in the Svp cells to sustain Svp cell fate. Thus, many of these early developmental factors have both positive and negative cross-regulatory effects upon downstream genes, although how these interactions occur mechanistically via identified enhancers is still being investigated. Moreover, some genes that are expressed in all cardioblasts have differential regulation depending upon if they are being expressed in a Svp cardioblast or a *tin* cardioblast. For instance, *Myocyte Enhancer Factor-2 (Mef2)* has separate regulatory elements for the two cell types [35]) and when a consensus binding site for the LIM homeodomain transcription factor Tailup is mutated in an enhancer of the basic helix-loop-helix transcription factor *Hand*, activity of the enhancer is only lost in the *Svp* cells [48].

There are also many genes expressed during cardioblast development that have not yet been characterized for a role in cardiac development. For example, *Transglutaminase (Tg)* is mainly expressed in the Svp cells, yet it requires *Mef2* for its expression. Ectopic *Mef2* expression does not strongly expand *Tg* on its own and *Mef2* is expressed in all cardioblasts, not just the Svp cells [49]. Therefore additional contributors must be involved in *Tg* activation in the Svp cells. Lastly, high throughput expression studies have identified additional genes expressed specifically in the Svp cells that have not been characterized yet, such as *CG8147* and *CG13196* [50]. It will be interesting to understand how these uncharacterized genes fit into the complex developmental network that leads to Svp cell specification and ostia development.

## 4. Genetic Control of Ostia Formation

Within the cardiac Svp cell population, unique patterns of gene expression must occur to promote inflow tract formation in the heart *versus* the aorta. While there are relatively few markers of ostia fate, the WNT gene *wingless* (*wg*) is expressed only in Svp cells of the heart [38]. Consistent with their roles in affecting *svp* expression, over-expression of either *abd-A* or *Ubx* results in expansion of expression of the Wingless signaling molecule into the aorta [37,38]. Over-expression of *Antp* also resulted in expansion of *wg* expression in the ectopically expressing *Svp* cells of the posterior aorta but not in the ectopic Svp cells of the anterior aorta [39]. During pupal development, *wg* is expressed in the Svp cells of the posterior aorta, signaling the metamorphosis of these cells into the adult ostia [26]. Thus, expression of *wg* depends upon Hox gene activity, and correlates closely with inflow tract formation, suggesting that it may play a critical role in inflow tract formation.

To test this hypothesis, Trujillo *et al.* [51] evaluated the role of *wg* and Wg signaling pathway members in formation of the ostia. Ostia are difficult to visualize, other than the elongated shape of the cells and their expression of *wg* and the alkaline phosphatase ortholog *CG8147*. All these features are absent in *abd-A* or *svp* mutant embryos, but are only reduced in *wg* mutants, suggesting a partial role for Wg in ostia cell development. Instead, another WNT ortholog, Wnt4, is also expressed in the ostia cells, and is required for normal ostia formation [52]. These factors function in an autocrine manner via the canonical Wg signaling pathway to promote ostia development. Interestingly, both groups demonstrate that ectopic Wg signaling in the aorta does not promote ectopic ostia fate [51,52], and other Hox-dependent factors must supplement Wg/Wnt4 signaling to direct the formation of ostia [51]. One possibility is that combined expression of *wg* and *Wnt4* is required to specify ectopic ostia. Factors controlling the formation of embryonic ostia are summarized in Figure 2C).

Understanding the genetic regulation of *wg* and *Wnt4* expression in the ostia will provide insight into the specification of these specialized cells. It has been shown that *wg* expression at the embryonic stage is dependent on *svp,* while over-expression of *svp* in the Tin cells of the heart will induce ectopic expression of *wg* in the presence of *abd-A* [38]. Similar experiments have not been carried out for *Wnt4*, nevertheless identification and dissection of the enhancers that control the expression of these two signaling molecules in the ostia will provide important mechanistic insight into how these cells are defined.

## 5. Formation of the Adult Ostia

At 30 h after puparium formation, the heart begins to remodel in response to a peak of the steroid hormone ecdysone [24]. The adult heart will form from cells spanning from the posterior border of T3 to A4 of the larval posterior aorta. Segment A5, which formed part of the unhistolyzed larval heart, will form the posterior end or terminal chamber of the adult heart [22,24,25,26]. In contrast to embryonic development, in the remodeling heart, UBX begins to be repressed in the contractile, Tin cells of the heart at 30 h APF and is only expressed in the Svp cells at 48hr APF. Monier *et al.* [24] found that knocking down *Ubx* resulted in loss of *wg* expression in the pupal Svp cells, suggesting that *Ubx* is required for adult ostia formation. This same result was obtained by knocking down Ecdysone signaling. In contrast, *abd-A* is only expressed in the terminal chamber of the adult heart and is only expressed in the two most posterior Svp cells of the terminal chamber [24]. Further evidence for a role for Wg/Wnt in adult ostia formation came from microarray studies in which the Wg signaling pathway was shown to be up-regulated in dissected pupal hearts of varying stages of development. To investigate these results, researchers analyzed the hearts of pupae expressing dominant negative forms of the Wg target genes *pangolin (pan)* and *disheveled (dsh),* and these animals failed to form ostia [53]. Taken together, a combination of Hox genes and signaling pathways are required for the specification of Svp cells and for ostia formation (Figure 2C).

Given that the ostia control entry of hemolymph into the cardiac tube, we have described them here as analogous to the vertebrate inflow tracts, corresponding to the atria. Genetic evidence in support of this assignment comes from the roles of the homologous factors Svp and COUP-TFII. In vertebrates, COUPT-TFII anchors a genetic program controlling atrium fate [54,55], similar to the requirement for Svp in specifying ostia fate in Drosophila inflow. Moreover, WNT2 and WNT4 are enriched in atrial tissue [55] and *WNT2*-expressing cells have been shown to contribute to the developing mouse inflow tract [56]. These roles are reminiscent of the requirements for *wg* and *Wnt4* in ostia formation in Drosophila. On the other hand, there are also similarities between formation of ostia and vertebrate heart valves: a Wnt signaling reporter is active in zebrafish valve precursors [57], and Wnt signaling is necessary for heart valve formation in zebrafish [58]. Moreover, the morphological changes in the ostia cells might be compared to the epithelial to mesenchymal transition of vertebrate heart valve progenitors into valve precursors [52]. As further details of the ostia specification and development processes are uncovered, a clear resolution of these points may be gained.

## 6. Regulatory Networks in Drosophila Valve Formation

Valve disease in humans affects on average 2.5% of the US population and its incidence increases with age [1]. The vertebrate heart contains aortic/pulmonary semilunar (SL) valves and mitral/tricuspid valves that separate the atria and ventricles [59]. *Drosophila* embryos and larvae possess one cardiovascular valve that separates the heart proper from the aorta, and in the adult there are three pairs of valve cells in segments A2 through A4 which separate the heart into four sections or chambers, each of which arise from a pair of Tin myocardial cells [22,53]. The purpose of both vertebrate and *Drosophila* valves is to promote unidirectional flow of blood or hemolymph [20,27]. Whereas vertebrate valves only allow blood to flow through them in one direction, hemolymph flow in Drosophila has been shown to be capable of reversal [25], thus vertebrate heart valves and the Drosophila cardiovascular valves must differ structurally from one another despite their functional similarities.

Currently, very little is known about the specification of Drosophila valve cells however, three studies have identified signaling pathways that are potentially required for their formation in adults. The first study, previously mentioned, identified an increase of transcripts of *PDGF and VEGF-receptor related (Pvr)* receptors as well as their ligands *PDGF and VEGF-related factor 2 (Pvf2)* during pupal development [53]. These genes are members of a receptor tyrosine kinase signaling pathway. When these authors analyzed the hearts of animals expressing a dominant negative form of *Pvr,* they saw a reduction in valve formation 20% of the time. Conversely, over-expression of *Pvr* resulted in ectopic valve formation 45% of the time. This mild phenotype suggests additional factors are involved in valve specification, but the findings nevertheless provides an inroad into the study of how Drosophila valve cells are specified. Further evidence supporting a role for *Pvr* in valve formation came from live imaging studies of the larval heart beat, where it was shown that *Pvr* mutants had defects in the mechanics of the heart beat [18].

In the third study, researchers investigated the role in adult heart development of an adaptor protein involved in the Wg signaling pathway, *pygopus (pygo).* Pygo has been shown to be part of a complex of Wg effectors (arm/β-Cat, lgs/BCL9 and pan/TCF) and is required in this complex for the activation of *Wg* targets [60,61,62,63]. Although Wg does not appear to be enriched at the locations of the adult valves, Tang *et al.* [27] investigated whether Pygo functioned downstream of *wg* in valve cell formation. Knockdown of *pygo* resulted in a loss of the characteristically dense organization of myofibrils at the valves, as well as an increase in the cardiac tube diameter where valve locations are normally narrower than neighboring cardiomyocytes. Over-expression of *pygo* rescued this phenotype but caused disorganization of the myofibrils. This suggests *pygo* has a role in valve formation, however null mutants for *pygo* had the normal number of cardiac nuclei suggesting it is not required for valve cell specification. When other Wg pathway genes were knocked down, such as *arm/β-Cat, lgs/BCL9 or pan/TCF*, there was only a slight increase in valve diameters. Double heterozygote mutants for the other *wg* components plus *pygo* did not have an increased valve diameter when compared to the effect of the single *pygo* heterozygote mutant. This might suggest that the contribution of *pygo* to valve formation is not through canonical *wg* signaling as has been demonstrated in zebrafish and mice [58,64] however, further investigation is needed. A summary of factors contributing to cardiovascular valve specification and development are summarized in Figure 2D.

A clear difference between vertebrate cardiac valves and their Drosophila counterparts is the relative size of the tissues. While vertebrate valves comprise hundreds of cells, the Drosophila valves each arise from a single pair of cells. Thus, many of the factors involved in maturation and growth of the vertebrate valves are not likely to be represented in the Drosophila system. However, there are still a number of genetic parallels between valve formation in vertebrates and Drosophila, in particular the involvement of WNT pathway components (orthologous to Drosophila Wg signaling) and VEGF signaling (orthologous to the Pvf2/Pvr pathway). VEGF is required for epithelial to mesenchymal transition of valve precursor cells and their subsequent morphogenesis [65]. Thus, Drosophila may be a useful model to understand the initial specification of valve cell fate.

## 7. Development and Specification of the Cardiac Outflow Tract

At the anterior of the Drosophila embryonic cardiac tube, the cardiac outflow tract is a complex structure that directs and stabilizes the cardiac outflow. Research from the Jagla laboratory has shown that the outflow tract arises from cells from at least three sources: invaginating cells from the head ectoderm migrate posteriorly and form a cluster of heart anchoring cells (HANCs); cardial cells from the anterior of the aorta contact the HANC cells; and cells arising from the pharyngeal mesoderm contact the cardial cells from an anterior and ventral location, contributing cardiac outflow muscle (COM) and an additional anterior cardioblast. These processes generate a stable structure that has a funnel-shaped appearance [66,67] Figure 1 inset.

A number of signaling molecules and transcription factors are important for formation of this structure. The Lb homeodomain transcription factors are expressed in a subset of the cardioblasts, and additionally in the migrating HANCs. Moreover, loss or suppression of *lb* function causes a failure of proper HANC migration, and a failure of the COM to contact the Lb cells of the anterior aorta [66]. In addition, the LIM homeodomain transcription factor Tailup is expressed in the aorta, the HANCs, and the COM, suggesting a role for this factor in formation of the outflow tract. Indeed, loss of Tup function causes a loss of Lb accumulation in the aorta, a failure of proper HANC migration, and inappropriate contact between the COM and the aorta cells [68].

The Slit/Robo signaling pathway also contributes to outflow tract formation. Each are expressed in both the anterior aorta and the HANCs, and loss of function in either or both tissues causes a failure of HANC migration and a lack of contact between the COM and the anterior aorta. Loss of function of the *D*E-Cadherin gene *shotgun* causes a similar phenotype [67]. Factors contributing to outflow tract specification and development are summarized in Figure 2E.

Clearly the Drosophila cardiac outflow tract is a complex structure that can serve as a model for how cells from three origins contribute to a specific structure. More importantly, these studies uncover intriguing similarities between the assembly of the Drosophila cardiac outflow tract and that of vertebrates. In vertebrates, recent studies have demonstrated that most of the right ventricle and much of the outflow tracts of the developing heart arise from a group of cells termed the second heart field [69], pioneered by the identification of second heart field cells based upon their expression of the Tup ortholog Islet-1. Just as with Drosophila Tup, Islet-1 mutants fail to form proper cardiac outflow structures [70]. Moreover, cells from the vertebrate neural crest contribute to outflow tract morphogenesis [71], similar to the role of the Lb-expressing HANC cells, that are of ectodermal origin. In addition, a subset of the cardiac neural crest cells express the Lb ortholog Lbx1, and Lbx1 is required for the specification of these cells [31]. Identification of additional factors that control outflow tract specification and morphogenesis in Drosophila therefore has the potential to provide significant new insight into second heart field development in vertebrates.

## 8. Regulatory Networks in Alary Muscle Formation

In addition to the contractile cells and inflow tract cells of the *Drosophila* dorsal vessel, there are multi-nucleated alary muscles that lie at the abdominal segment borders. These muscles attach dorsally close to a subset of *svp*-expressing pericardial cells via a network of extracellular matrix fibers, and laterally to the cuticle [19,20,72]. It has been suggested that these muscle cells support the heart structurally and contract along with it to facilitate the influx of hemolymph to the interior of the vessel [16,73]. These specialized muscle cells make contact with several body wall muscles, the tracheal system, tips of the Malphigian tubules and anchor to the epidermal tendon cells [19,20,73,74]. Finally, they are attachment sites for neurons during post-embryonic stages [74]). From a functional viewpoint, alary muscles could conceivably be compared to the various ligaments in the vertebrate heart which anchor it to the diaphragm, spinal cord and sternum [75].

Alary muscle patterning is dependent upon two *Bithorax Complex* genes [19], where *Ubx* is expressed in the anterior alary muscles that attach to the aorta, and *abd*-A is expressed in the more posterior cells that attach to the heart. Alary muscles that express *Ubx* do not form in *Ubx* mutants, whereas in *abd-A* mutants, the normal number of alary muscles form because *Ubx* expression expands posteriorly in *abd-A* mutants. In *Ubx abd-A* double mutants no alary muscles form, and in the converse experiments, in which either of these genes is over-expressed, supernumerary alary muscles form and attach to the anterior aorta. Clearly, Hox gene function is essential to the specification of these cells.

The alary muscle progenitor cell was first identified at embryonic stage 11 by expression of *optomotor-blind-related-gene-1 (org-1)*, a *T-box* transcription factor [76]. This progenitor also expresses *tup*, the ortholog of the LIM-homeodomain gene *Islet-1* [21,72]. The alary progenitors lie posterior to the DA2 adult muscle precursor progenitor cells and divide at stage 12 into one cell that continues to express *org-1* and a sister cell that is transiently positive for *Zfh1*, a general adult muscle precursor marker. Using time-lapse imaging, researchers followed the differentiation of each of the alary muscle founder cells and demonstrated that they produce filopodial extensions, fuse with myoblasts, form syncytial fibers and finally attach to the heart and lateral epidermis [72].

In both *tup* and *org-1* mutants, alary muscles are either missing or severely deformed [72,76]. *tup* expression is lost in many of the muscles in *org-1* mutants, and over-expression of *org-1* causes alary muscle thickening, an increase in *tup* expressing nuclei and in some segments, a duplicated alary muscle. The activation of *tup* by *org-1* is direct [72] but only occurs in either the alary muscle founder cell or its sibling cell, with ectopic expression experiments suggesting additional activators or removal of repressors are required for *tup* transcription. Clearly there is a unique regulatory program that specifies these cells, summarized in Figure 2F, and that might be recapitulated in higher animals to form related structures.

## 9. Regulatory Networks in the Formation of Ventral Longitudinal Muscle Fibers in the Adult Heart

During the pupal remodeling of the heart, imaginal cells migrate over the persisting larval heart and form ventral longitudinal muscles (VLMs) [20,22]. These imaginal cells are separate from the *tin/svp* expressing cardiomyocytes discussed thus far. While an initial report suggested that the ventral fibers arose from a subset of lymph gland cells [26], more detailed recent studies have instead demonstrated that the ventral fibers arise from the transdifferentiation of three anterior pairs of alary muscles [77]. Schaub *et al.* found that after pupal stage P4, these muscles began separating into mononucleated mesenchymal cells which they termed AMDCs (alary muscle-derived cells). As development proceeds, the AMDCs began to establish contact via cellular protrusions across the ventral side of the heart and eventually fused with each other and dorsal adult muscle precursors to form syncytia which eventually form the multinucleated VLMs. Interestingly, they demonstrated that the same transcription factors required for alary muscle formation (the vertebrate Tbx1 and Islet1 homologs Org-1 and Tailup) are required for alary to VLM transformation. Specifically, knockdown of *org-1* prevented the dedifferentiation of alary muscles and loss of VLM formation while knockdown of *tup* prevented alary muscle transdifferention with an occasional stunted VLM forming. Schaub *et al.* discovered that the homeotic selector gene *Ubx* was expressed in the three anterior alary muscles during pupariation while *abd-A* was restricted to the posterior four sets. When they suppressed *Ubx* by over-expression of *abd-A*, no VLMs formed and additional AMs developed. In addition, activity of an *Org1* enhancer was lost, suggesting that UBX might regulate *org-1* directly.

Two additional studies demonstrated a loss of VLM formation when either the Ecdysone Receptor [24] or Heartless (an FGF receptor) were inactivated ([53]. Investigating this in more depth, Schaub *et al.* [77] found that loss of EcR blocked alary muscle de-differentiation and transdifferentiation, while loss of the FGF pathway only prevented transdifferentiation along with a reduced number of total muscle fibers. Similar to the results of blocking the FGF pathway, the alary muscles of late mutants of NK-homeodomain transcription factor gene *tinman* (*tin)* were able to de-differentiate, however migration and transdifferentiation were prevented. Finally, knock down of the muscle transcription factor *Myocyte Enhancer Factor 2 (Mef2)* also resulted in a reduction in VLM formation. Previous work has demonstrated differential expression of actin gene family members in the heart. The VLMs express the major embryonic actin, *Actin 57B* (*Act57B)* whereas *Actin 87E* is not expressed in the longitudinal cells but is expressed in the cardiac tube of the adult [26]. *Mef2* has been shown to be a direct activator of *Act57B* during embryogenesis [78] and it would be a reasonable conclusion that MEF2 may also activate *Act57B* in the VLMs. A summary of the factors controlling VLM formation is presented in Figure 2G.

The elongation of the vertebrate heart tube and formation of the outflow tract depends upon progenitor cells from the pharyngeal mesoderm termed second heart field and requires *Tbx1*, *Isl1, FGF* signaling and *mef2C* among other factors [79,80]. The process of VLM formation in *Drosophila* could be seen as similar to that in vertebrates, due to its utilization of progenitors from neighboring alary muscles. In this process, muscles transform and develop onto an existing heart tube. While the VLMs and the vertebrate outflow tract might not necessarily be homologous structures, the regulatory factors that direct the process of expanding upon a simple organ into a more complex structure show striking similarities.

## 10. Conclusions

Overall, these findings uncover a compelling homology across species in the molecular mechanisms not just of cardiac specification but also of the formation of individual cell types within the heart. Just as the Drosophila system was central to uncovering the molecular mechanisms by which the cardiac field is specified, this same system is poised to uncover new genetic processes that will inform vertebrate studies on valve development, inflow tract formation, and the second heart field. Given the small size of some of the structures being investigated, a challenge in the future will be to develop further tools that enable the visualization and analysis of the specialized cell types of the Drosophila heart.
